# Optimization of “vehicle-UAV” joint distribution routing for cold chain logistics considering risk of epidemic spreading and green cost

**DOI:** 10.1371/journal.pone.0306127

**Published:** 2024-06-26

**Authors:** Gang Liu, Qian Liu, Hao Guo, Ming Xiang, Jinyan Sang

**Affiliations:** 1 Research Center of Hubei Logistics Development, Hubei University of Economics, Wuhan, China; 2 Collaborative Innovation Center for Emissions Trading System Co-constructed by the Province and Ministry, Hubei University of Economics, Wuhan, China; 3 School of Business Administration, Hubei University of Economics, Wuhan, China; 4 School of Management, Wuhan Textile University, Wuhan, China; 5 Hubei Enterprise Culture Research Center, Hubei University of Economics, Wuhan, China; 6 School of Statistics and Mathematics, Hubei University of Economics, Wuhan, China; National Taiwan University of Science and Technology, TAIWAN

## Abstract

To address the epidemic, such as COVID-19, the government may implement the home quarantine policy for the infected residents. The logistics company is required to control the risk of epidemic spreading while delivering goods to residents. In this case, the logistics company often uses vehicles and unmanned aerial vehicles (UAVs) for delivery. This paper studies the distribution issue of cold chain logistics by integrating UAV logistics with epidemic risk management innovatively. At first, a "vehicle-UAV" joint distribution mode including vehicles, small UAVs and large UAVs, is proposed. The green cost for vehicles and UAVs is calculated, respectively. The formula for infection risk due to large numbers of residents gathering at distribution centers to pick up goods is then derived. Furthermore, based on the control of infection risk, an optimization model is developed to minimize the total logistics cost. A modified ant colony algorithm is designed to solve the model. The numerical results show that the maximum acceptable risk and the crowd management level of distribution centers both have significant effects on the distribution network, logistics cost and number of new infections. Our study provides a new management method and technical idea for ensuring the needs of residents during the epidemic.

## 1 Introduction

In the context of accelerating quality upgrading in the consumer market, people’s quality requirements for fresh goods have gradually increased, and market demand has continued to expand, which has led to the rapid development of cold chain logistics. The normal operation of logistics system is an indispensable part of social sustainability. However, the outbreak of COVID-19 epidemic made the cold chain logistics and distribution of fresh goods face extremely severe challenges. Under the emergence of the epidemic, "national online grab vegetables" led to a surge in demand for fresh goods, and online orders needed to be guaranteed by offline cold chain logistics distribution. Traditionally, the fresh goods are carried by vehicles to the distribution center and further delivered to the residents waiting here. During this process, face-to-face contact exists widely, increasing the risk of cross-infection between deliveryman and community residents. As a result, there may be more infected residents and even more deaths. Therefore, the traditional distribution mechanism cannot well solve the problem of fresh distribution under the background of sudden epidemic [1]. The sustainable development of society is under the threat of COVID-19 pandemic.

As a new tool, unmanned aerial vehicle (UAV) has achieved rapid development in the field of logistics and transportation during the past few years of COVID-19 pandemic. Particularly, during the COVID-19 pandemic, the Chinese government implemented the home quarantine policy for epidemic areas with traffic control and travel restriction. Many infected residents were put in quarantine timely and had to wait for the home delivery service offered by the logistics company. Furthermore, the logistics company was ordered to control the risk of epidemic spreading while delivering goods to residents. As a result, many companies introduced the "vehicle-UAV" joint operation service mode, which performed the "non-contact" distribution service effectively. On one hand, this mode can alleviate the shortage of distribution personnel caused by the epidemic and reduce the risk of crowd infection in the process of residents picking up their own goods. On the other hand, it can avoid the high logistics distribution cost and huge value loss in the transportation process of fresh goods. In fact, vehicles and UAVs were used together in the cold-chain logistics of many Chinese cities during the COVID-19 pandemic. The optimization of "vehicle-UAV" joint distribution scheme is valuable for reducing the cost of the logistics company and controlling the risk of epidemic spreading.

Therefore, this paper focuses on the cold chain logistics distribution of fresh goods using "vehicle-UAV" joint operation in the context of the epidemic. The main contributions are: (1) In view of the infection risk caused by the gathering of some residents at the pick-up point, this paper uses the SI modeling method of infectious disease to derive the analytical formula of risk, and takes the control of infection risk as the basis for optimal selection of distribution plan. (2) Our study integrates UAV logistics with epidemic risk management, providing a new management method and technical idea for ensuring the needs of residents during the epidemic. It promotes the application of UAV technology in the field of fresh logistics and epidemic risk infection management. (3) Based on the differences in size, load and endurance, this paper divides UAVs into small UAVs and large UAVs, and studies the "vehicle-small UAVs—large UAVs" fresh cooperative delivery operation during the epidemic period. The comparison between this paper and existing literature can be found in Table 1. The results of this paper provide new ideas for improving the efficiency of fresh distribution, timely meeting the demand for fresh commodities of residents and reducing the risk of epidemic transmission during the epidemic period. As far as we know, this is the first research work to study the optimization of "vehicle-drone" joint distribution scheme from the quantitative perspective of controlling the risk of epidemic spreading.

**Table 1:** Main differences between our study and the existing literature.

references	“Vehicle-UAV” joint distribution	different types of UAVs	green cost	home quarantine policy during the epidemic	quantitative analysis of contagion risk
[7, 13–17]	Χ	Χ	√	Χ	Χ
[23–29]	√	Χ	Χ	Χ	Χ
[31, 33]	Χ	Χ	Χ	√	Χ
[30, 32, 34]	√	Χ	Χ	√	Χ
This paper	√	√	√	√	√

The rest of this article is organized as follows. Section 2 introduces the literature review of LRP and "vehicle-UAV" joint operations in cold chain logistics under the background of epidemic. The third part discusses the construction of the "vehicle-UAV" integrated logistics optimization distribution model for fresh goods under the control of the spread risk of the epidemic. Section 4 introduces the improved ant colony algorithm to solve the model. A numerical example is given in Section 5. Finally, some conclusions are drawn in section 6.

## 2 Literature review

Location-routing problem (LRP) is a classical combinatorial optimization problem, which needs to determine both the location of candidate facilities and the path of vehicles [2]. Since Cooper [3] first proposed LRP, it has gradually become a hot issue in the fields of supply chain, logistics and operation research optimization. Location-path planning has been applied in many fields, such as waste recycling, emergency logistics, low-carbon, closed-loop supply chain, etc. For example, Li et al. [4] developed a novel multi-objective optimization model for the LRP for biomass waste collection using tailoring evolutionary algorithm. Adarang H et al. [5] addressed a location-routing problem (LRP) under uncertainty for providing emergency medical services (EMS) during disasters, which is formulated using a robust optimization (RO) approach. Jiang et al. [6] established a low-carbon open location- routing problem model related to distribution center size and distribution path, and designed a quantum evolutionary algorithm to solve it. Govindan et al. [7] studied the location-inventory-routing problem to design a circular closed-loop supply chain network with carbon tax policy for achieving circular economy. Guo et al. [8, 9] not only considered the location-inventory decisions for closed-loop supply chain management in the presence of the secondary market, but also solved a multi-commodity location-inventory problem in a closed-loop supply chain with commercial product returns. At the same time, location-path planning has also been introduced into the fresh cold chain industry. In recent years, scholars have conducted a lot of research in the field of LRP of fresh commodity, mainly considering the influence of product perishability, time window limit, low-carbon environmental protection and other factors on the model. Rahmanifar et al. [10] designed a novel nonlinear multi-objective model designed to concurrently optimize warehouse facility location and vehicle routing, addressing the challenges inherent in cold chain logistics processes. Moghaddasi et al. [11] developed a mixed integer nonlinear programming routing-location model to improve the cold chain logistics network design within the Balanced Score Card pillars by maximizing the customer satisfaction and minimizing unit cost and greenhouse gas emissions in order to solve the optimization problem of the marine product distribution logistics system. Ma et al. [12] established a multi-agent optimization model of fresh agricultural distribution location—route based on conflict cooperation, considering the high distribution cost of fresh agricultural products and high product loss, and took into account customer fuzzy time window in the model. Li et al. [13] built a multi-vehicle cold chain logistics vehicle routing optimization model with carbon emission cost and time window on the basis of considering the congestion index. Govindan et al. [14] proposed a multi-objective optimization model for the network distribution of perishable food supply chain considering the time window constraints, and studied the cold chain location path from the perspective of low carbon and environmental protection, so as to minimize the impact of carbon emissions on the environment. From the perspective of low-carbon and environmental protection, Wang et al. [15] considered the carbon footprint of cold chain logistics location path optimization according to the characteristics of perishable products. Leng L et al. [16] analyzed the low-carbon LRP of cold chain logistics, established a dual-objective model with the minimization of total logistics cost and vehicle waiting time, and selected a variety of algorithms for solution comparison. Pan [17] established a cold chain logistics route optimization model from the perspective of low-carbon, and solved the model by using Wolf pack algorithm and ant colony algorithm. Mohammadi et al. [18] developed a multi-objective mixed-integer non-linear programming model for a four-level sustainable supply chain of a perishable product with price-dependent demand and deterioration rates. In addition, some scholars have carried out extended studies on the influence of factors such as facility capacity limitation [19], influence of multiple vehicle types [20], and inventory cost [21, 22] on LRP. The above literature indicates that considering the influence of location problem in fresh distribution route planning can effectively reduce the overall logistics distribution cost.

In view of the particularity of the epidemic background, vehicles cannot directly complete the distribution service for isolated residents, and the collaborative distribution characteristics of vehicle-UAV should be considered in the path planning process. As for the research on vehicle-UAV cooperative distribution, Murray and Chu [23] earlier proposed the traveling salesman problem of truck and UAV cooperative distribution. Aiming at the optimal route and scheduling of UAV and delivery truck, they proposed a mixed integer linear programming model for two UAV delivery problems. This new package delivery paradigm facilitates last-mile delivery. At present, the research on vehicle-UAV collaborative delivery is mainly differentiated from the launch and landing position selection of UAVs, the number of UAVs that vehicles can carry, and the collaborative operation mode of UAVs. For example, Carlsson and Song [24] consider that the UAV takes a package from the truck, and at the same time, the truck continues its route. After delivering the package, the UAV returns to the truck to take another package, and the vehicle and the UAV can meet at any position. Chang and Lee [25] proposed a new method of nonlinear programming model, which used K-means clustering to group customers and determine the UAV launch location, so as to expand the UAV distribution area and reduce the driving route of vehicles. Boysen et al. [26] considered the situation that a truck could carry multiple UAVs, distinguished whether multiple UAVs or one UAV were placed on the truck, and whether the take-off and landing stations must be the same. Karak et al. [27] proposed a mathematical formula and an effective solution for the hybrid vehicle-drone routing problem for pick-up and delivery services, considering that vehicles only serve as the carrier of UAVs and all delivery services are completed only by UAVs. Ham [28] did not regard vehicles as UAV carriers, and UAV and trucks were distributed in parallel, and studied the comprehensive scheduling of multiple warehouses carrying truck fleets and UAV fleets to achieve excellent operation. Moshref-Javadi et al. [29] considered the load capacity and flight time limit of UAV. Although some research results have been achieved on the problem of vehicle-UAV collaborative delivery, there is a lack of research and discussion on the effective cooperation of "vehicle-UAV" and the provision of distribution services for normal and isolated residents under the epidemic environment by setting vehicle temperature control. Gao et al. [30] studied the problem of coordinated distribution of emergency resources between unmanned ground vehicles (UGVs) and unmanned aerial vehicles (UAVs) based on nested vehicle paths, including two sub-problems: how to accept the commander’s operation order and how to generate nested vehicle routing planning.

In addition, a small number of literatures have carried out research on the location and routing problem of fresh food under the background of epidemic. For example, Li et al. [31] studied the retail logistics distribution vehicle routing problem considering the order release time under the emergency epidemic environment. The results show that the model and algorithm can provide effective decision support for the efficiency improvement and cost control of retail logistics distribution. Zhang and Li [32], in order to effectively solve the "contact-free" distribution problem of fresh commodities under the current epidemic background, proposed the site-routing optimization problem of fresh distribution based on the epidemic background, taking into account the impact of the epidemic, commodity perishability, temperature control characteristics, site selection and vehicle- UAV collaborative distribution route planning. Shibu and Philip [33] considered the context of COVID-19 and proposed a distribution system using UAVs, in which delivery agencies and customers work together to distribute goods, and established a new framework to find the optimal path of delivery UAVs. Based on the practical experience of COVID-19 vaccine delivery in China, Zheng et al. [34] proposed a hybrid approach of machine learning and evolutionary computing, using an evolutionary algorithm to determine vehicle routes and distribute vaccines daily from satellite/warehouse to vaccination points.

However, there are the several shortcomings in the existing literatures: (1) The impact of crowd infection risk on the "vehicle-UAV" delivery path, cost and number of infected people during the epidemic is not considered from a quantitative perspective. (2) There is lack of research about the effect of home quarantine policy on the distribution scheme. (3) The studies of "vehicle-UAV" logistics distribution during a major epidemic in the literature has several defects in the size setting of UAV, such as not considering the coordination of large and small UAVs, and the restrictions on load and endurance. (4) There is lack of research on the location and path of fresh distribution based on "vehicle-UAV" collaborative distribution under the impact of the epidemic.

In view of this, this paper focuses on the cold chain logistics distribution of fresh commodities under the home quarantine policy of an emergency epidemic and studies the risk of mass infection and logistics distribution network under the joint operation of "vehicle-UAV". The main differences between our study and the existing literature are summarized in Table 1.

## 3. Model framework

### 3.1 Problem description

The epidemic, which spreads through the air (COVID-19), and the home quarantine policy are considered throughout this paper. If some resident is infected and shows the symptoms, he will be sent home and put in quarantine timely.

**Fig 1:**
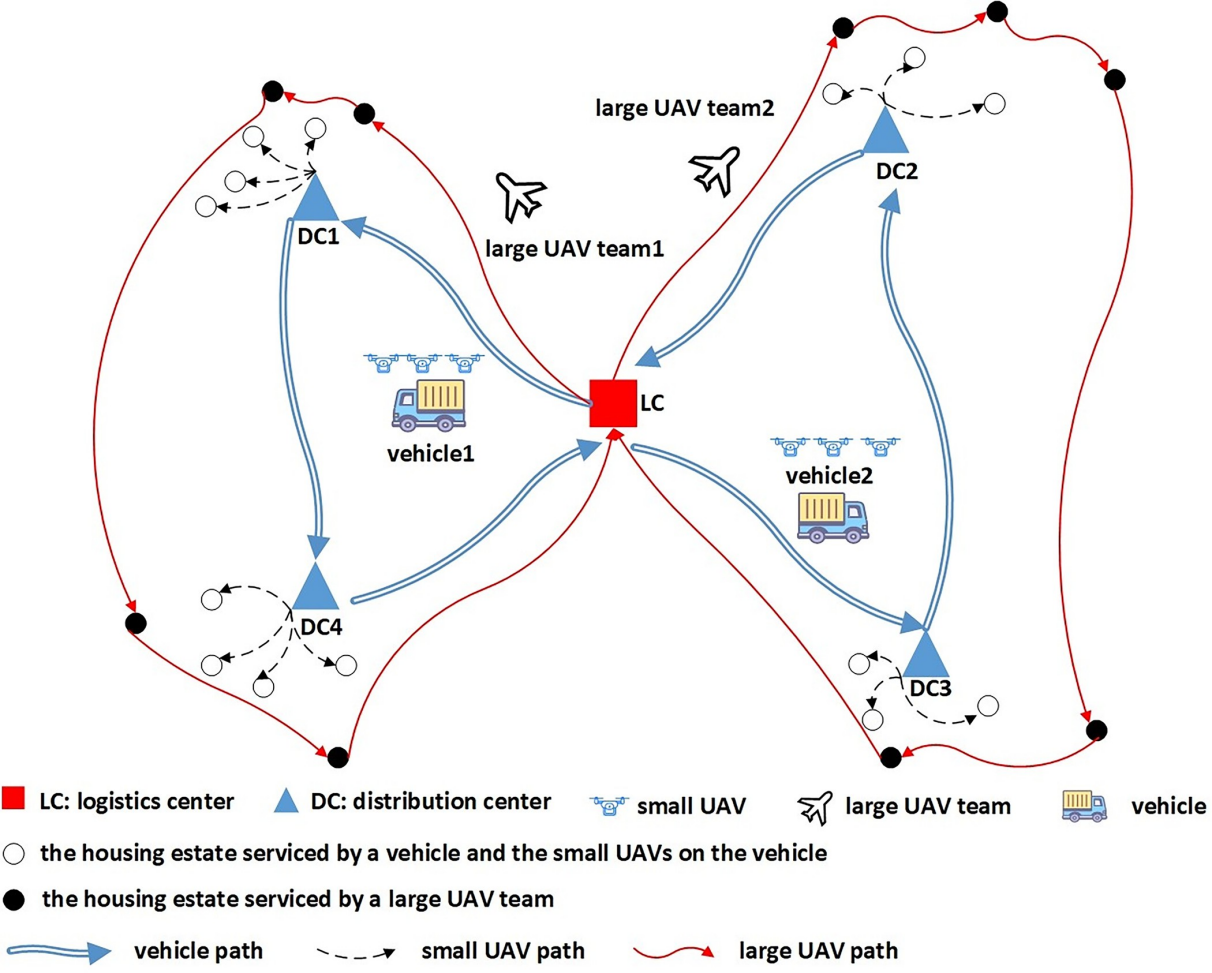
The location-routing logistics network of fresh green distribution in the epidemic environment.

**Fig 2:**
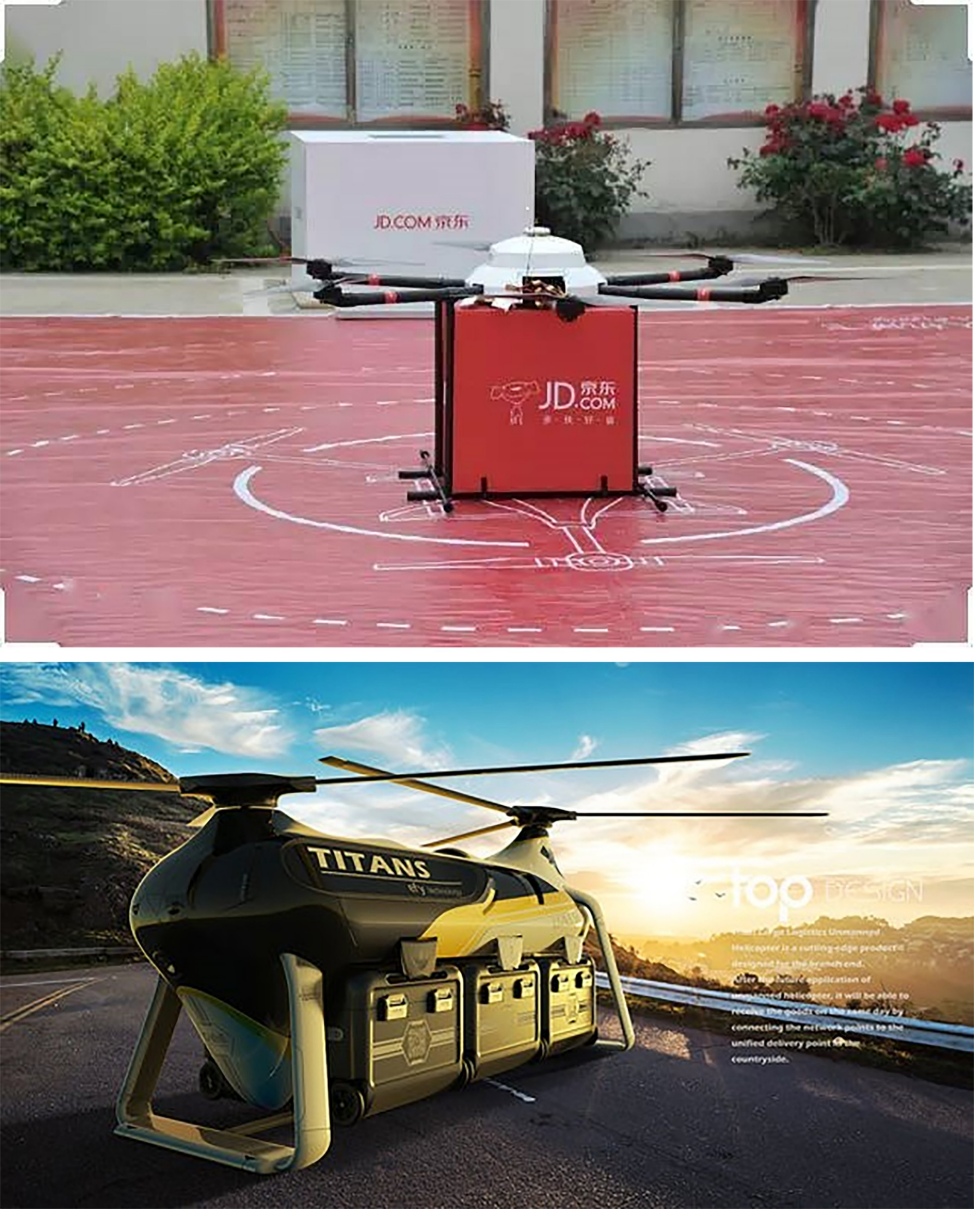
(a) the small UAV; (b) the large UAV.

A logistics company plans to deliver the goods to residents, who live in a great number of housing estates. The company can use two distribution methods—vehicle and UAV. The location-routing logistics network of fresh green distribution in the epidemic environment considered in this paper is shown in Fig 1. It can be seen that, there are several distribution centers and the unique logistics center. Every housing estate falls within the scope of its unique distribution center and the vehicle can provide services to the residents only through the distribution center. The vehicle carries the goods from the logistics center to a series of distribution centers and returns to the logistics center finally. When the vehicle arrives at a distribution center, the goods are unloaded and sent to the residents waiting here. If a small number of infected residents are put in quarantine at home, several small UAVs (Fig 2A) carried by the vehicle can send the goods to those residents directly. For the residents waiting at the distribution center, some of them may be infected but asymptomatic and they may spread the virus to others unintentionally. As a result, the risk of epidemic spreading rises. Moreover, if there are many infected residents in a housing estate, the company can send a team of large UAVs (Fig 2B) to deliver goods. The large UAV flies from the logistics center to some housing estates and returns to the center finally. Because it is non-contact, the UAV delivery does not result in more infections.

This paper aims to study how the logistics company makes the optimal decision about the integrated “Vehicle-UAV” distribution network with controlling the risk of epidemic spreading. The assumptions about the studies on the spread of epidemics in this paper, which mainly stems from the classical SI epidemic model, are given as follows.

### The main assumptions about the spread of epidemics

A1. The population is divided into infected people and uninfected people, and the total population remains unchanged.
A2. It takes no more than one day for a logistics company to distribute goods in the city, and it takes significantly more than one day for an infected person to be cured. Therefore, this paper does not consider the situation that the infected person is cured and becomes healthy again.
A3. A healthy person becomes infected with the virus by coming into close contact with an infected person while waiting in line at a distribution center to pick up goods. Eventually, the epidemic spread.
A4. The rate at which a population is infected with the virus is proportional to the base rate of infection, the average number of people each infected person comes into contact with per unit time, the probability of a single infected person coming into contact with a healthy individual, and the number of infected people.

The above assumptions will help us model the spread of epidemics in distribution logistics, calculate the number of new infections and evaluate the risk of epidemic spreading, as shown by Section 3.3.2.

### 3.2 Model parameters and variables

According to the needs to build the model, this paper sets the following parameters and variables, as shown in Table 2.

**Table 2:** The explanation of parameters and variables.

Parameters and Variables	Meaning
*0*	The unique logistics center station
*N*	The set of distribution centers
*N*_0_	*N*_0_ = *N*∪0 is the set of all distribution centers processing freights
*S*	The set of housing estates
*S*(*n*)	The set of housing estates belonging to the scope of service of distribution center *n*, *S*(*n*)∩*S*(*m*) = *ϕ* for *n*≠*m*
*K*	The set of delivery vehicles
*W*	The set of teams of large UAVs
*E*_*n*_	The capacity of distribution center *n*
*q*_*s*_	The total demand of housing estate *s*
*α*_*s*_∈(0,1)	The percentage of infected residents in housing estate *s* who are symptomatic and have been sent home and put in quarantine
${\hat{\alpha }}_{s}\in (0,1)$	The percentage of infected residents in housing estate *s* who are asymptomatic, yet to be discovered by epidemic prevention workers, free to move and infectious
$\bar{Q^{k}}$	The capacity of vehicle *k*
$t_{nm}^{V}$	The time of a vehicle traveling from center *n* to center *m*
$\bar{D^{w}}$	The capacity of team *w* of large UAVs
$t_{sj}^{U}$	The time of a team of large UAVs flying from housing estate *s* to housing estate *j*
*β*_*n*_	The average number of persons that a resident closely contacts when he is waiting at center *n* to get his own goods
*δ*	Incidence of the virus
$c_{k}^{V}$	The unit fixed cost of vehicle *k*
$c_{w}^{U}$	The unit fixed cost of large UAV team *w*
*d*_*nm*_	The distance between *m* and *n*
*c*_*fuel*_	Fuel price
*c*_*tax*_	Carbon tax
*σ*_*i*_	Fuel emission coefficient, *i*∈{vehicle,large UAV}
*c*_*r*_	Unit refrigeration cost
*LT*_*s*_	Latest arrival time of housing estate *s* to get the goods
*θ*	Unit delay penalty cost
*e*_*s*_	If housing estate *s* is serviced by some vehicle, *e*_*s*_ = 1; if *s* is serviced by large UAVs, *e*_*s*_ = 0
*y*_*ns*_	If housing estate *s* is serviced by a vehicle passing through center *n*, *y*_*ns*_ = 1; if *s* is serviced by large UAVs, *y*_*ns*_ = 0
$f_{n}^{k}$	If vehicle *k* passes through center *n*, $f_{n}^{k}=1$; otherwise $f_{n}^{k}=0$
$x_{nm}^{k}$	If vehicle *k* passes from distribution center *n* to distribution center *m*, $x_{nm}^{k}=1$; otherwise $x_{nm}^{k}=0$
$h_{s}^{w}$	If housing estate *s* is serviced by team *w* of large UAVs, $h_{s}^{w}=1$; otherwise $h_{s}^{w}=0$
$p_{sj}^{w}$	If team *w* of large UAVs passes from housing estate *s* to housing estate *j*, $p_{sj}^{w}=1$; otherwise $p_{sj}^{w}=0$
*r*_*n*_	The total goods received by distribution center *n*
$Q_{0}^{k}$	The goods weight of vehicle *k* starting from logistics center *0*
$Q_{m}^{k}$	The goods weight of vehicle *k* just arriving at center *m*
$t_{n}^{k}$	The time of vehicle *k* arriving at center *n*
$T_{n}^{k}$	The time that vehicle *k* needs to stay at center *n*
$D_{0}^{w}$	The goods weight of team *w* of large UAVs starting from logistics center station *0*
$D_{s}^{w}$	The goods weight of team *w* of large UAVs just arriving at housing estate *s*
$t_{s}^{w}$	The time of team *k* of large UAVs arriving at housing estate *s*
*T*_*s*_	The time that team *w* of large UAVs needs to stay at housing estate *s*
${\bar{t}}_{n}$	The average waiting time of residents at center *n* to get the goods
*R*_max_	The maximum acceptable risk, which is the acceptable upper limit for the number of new infections caused by residents gathering at a distribution center to pick up goods

### 3.3 Model design

#### 3.3.1 Objective function analysis of model

*1*. *Fixed costs*. The fixed costs mainly conclude the operation, maintenance and labor costs [15, 35]. The fixed cost of vehicles is

$$C_{1}^{V}=\sum_{k\in K}\sum_{m\in N}c_{k}^{V}x_{0m}^{k}$$ (1)

where $c_{k}^{V}$ includes the related cost of the small UAVs going with a vehicle. Similarly, the fixed cost of large UAVs is

$$C_{1}^{U}=\sum_{w\in W}\sum_{s\in S}c_{w}^{U}p_{0s}^{w}$$ (2)

Then the total fixed cost is

$$C_{1}=\sum_{k\in K}\sum_{m\in N}c_{k}^{V}x_{0m}^{k}+\sum_{w\in W}\sum_{s\in S}c_{w}^{U}p_{0s}^{w}$$ (3)

*2*. *Green cost*. The green cost equals to the fuel cost plus the carbon tax, which further depend on the amount of fuel consumption. According to the literature [36–40], the fuel consumption of vehicles is

$$F^{V}=\sum_{k\in K}\sum_{n\in N_{0}}\sum_{m\in N_{0}}x_{nm}^{k}\rho (Q_{n}^{k}-r_{n})d_{nm}$$ (4)

where

$$\rho (M)=\rho_{0}+\frac{\rho_{*}-\rho_{0}}{Q}M$$ (5)

is the fuel consumption per unit distance of a vehicle. The fuel cost and the carbon tax for all vehicles are respectively

$$FC^{V}=c_{fuel}\sum_{k\in K}\sum_{n\in N_{0}}\sum_{m\in N_{0}}x_{nm}^{k}\rho (Q_{n}^{k}-r_{n})d_{nm}$$ (6)

$$TC^{V}=c_{tax}\sigma \sum_{k\in K}\sum_{n\in N_{0}}\sum_{m\in N_{0}}x_{nm}^{k}\rho (Q_{n}^{k}-r_{n})d_{nm}$$ (7)

Thus, the green cost for vehicles is

$$GC^{V}=\left(c_{fuel}+c_{tax}\sigma \right)\sum_{k\in K}\sum_{n\in N_{0}}\sum_{m\in N_{0}}x_{nm}^{k}\rho (Q_{n}^{k}-r_{n})d_{nm}$$ (8)

Similarly, the green cost for large UAVs is

$$GC^{U}=\left(c_{fuel}+c_{tax}\sigma_{U}\right)\sum_{w\in W}\sum_{i\in S_{0}}\sum_{j\in S_{0}}p_{ij}^{w}\mu (D_{i}^{w}-q_{i})d_{ij}$$ (9)

where

$$\mu (M)=\mu_{0}+\frac{\mu_{*}-\mu_{0}}{D}M$$ (10)

The total green cost of logistics system is

$$C_{2}=\left(c_{fuel}+c_{tax}\sigma_{V}\right)\sum_{k\in K}\sum_{n\in N_{0}}\sum_{m\in N_{0}}x_{nm}^{k}\rho (Q_{n}^{k}-r_{n})d_{nm}+\left(c_{fuel}+c_{tax}\sigma_{U}\right)\sum_{w\in W}\sum_{i\in S_{0}}\sum_{j\in S_{0}}p_{ij}^{w}\mu (D_{i}^{w}-q_{i})d_{ij}$$ (11)

*3*. *Refrigeration cost*. Vehicle and large UAV are the transportation facilities of logistics system. However, most of the large UAVs being used within the city do not equip with a refrigerator because of the limited size and rapid delivery speed. Therefore, the refrigeration cost is only related to the vehicles. It is

$$C_{3}=c_{r}\sum_{k\in K}\sum_{n\in N}t_{n}^{k}f_{n}^{k}$$ (12)

*4*. *Delay penalty cost*. During the epidemic, the residents who are infected with the virus and have symptoms of infection are required to stay at home, while other residents can walk to the given distribution center and get the goods. Due to the seriousness of epidemic, logistics companies must deliver the goods to the specified center within the required time; otherwise, they have to pay for the delay penalty cost. When vehicle *k* arrives at the center *n* at time $t_{n}^{k}$, an asymptomatic resident has to walk from their housing estate s to center *n* and wait about ${\bar{t}}_{n}$. After all these, this resident gets his goods. Thus, the delay penalty cost is

$$C_{4}=\sum_{k\in K}\sum_{n\in N}\sum_{s\in S(n)}f_{n}^{k}\theta \max\{t_{n}^{k}+\frac{d_{sn}}{v_{walk}}+{\bar{t}}_{n}-LT_{s},\;0\}$$ (13)

where $\max\{t_{n}^{k}+\frac{d_{sn}}{v_{walk}}+{\bar{t}}_{n}-LT_{s},\;0\}$ is the delay time for vehicle *k* to service distribution center *n*, *d*_*sn*_ is the distance between *n* and housing estate, *v*_*walk*_ is the average walking speed of residents and *LT*_*s*_ is the latest demand service time for housing estate *s*. Moreover, the time ${\bar{t}}_{n}$ can be computed as

$${\bar{t}}_{n}=\sum_{k\in K}\sum_{s\in S(n)}\frac{1}{v_{1}}(1-\alpha_{s})q_{s}y_{ns}f_{n}^{k}$$ (14)

where *v*_1_ is the average pick-up speed of residents, $\sum_{k\in K}\sum_{s\in S(n)}(1-\alpha_{s})q_{s}y_{ns}f_{n}^{k}$ is the quantity of goods which belong to the asymptomatic residents waiting at center *n*. Moreover, because the large UAV has a much faster speed than vehicles in reality, we do not consider the delay case of large UAVs.

#### 3.3.2 Risk analysis of the residents waiting for goods at the distribution center

The risk of infection stems from the close contact among residents who get together at the distribution center and wait in lines to get their goods. It is because there may be infected but asymptomatic residents in the crowd. Section 3.3.2 aims to quantify the risk of infection, helping us design reasonable constraint conditions later. Throughout this paper, the risk of a distribution center is denoted as its number of new infections. According to the classical SI model [41], the change of total infections in center *n* over time is

$$\frac{dI_{n}}{dt}=\delta \beta_{n}\frac{I_{n}(N_{n}-I_{n})}{N_{n}}$$ (15)

where *δ* is the infection rate when a healthy resident has close contact with one infector, *β*_*n*_ is the average number of persons that a resident closely contacts when he is waiting at center *n* to get the goods, *I*_*n*_ and *N*_*n*_ denote the number of infection and the number of residents getting together at center *n*, respectively. It is clear that, *δ* will decrease if more residents are persuaded by managers at center *n* to wearing masks, and *β*_*n*_ will decline if more residents are commanded to keep distance with each other while waiting in line at center *n*. Thus, overall infection rate *δβ*_*n*_ measures the management level of center *n*. The management level increases as *δ* and *β*_*n*_ decrease. In addition, $\frac{N_{n}-I_{n}}{N_{n}}$ in Eq (15) is the probability of a single infected person coming into contact with a healthy individual. Eq (15) means that the number of new infections, i.e., the risk of distribution center *n*, is

$$Risk_{n}=\Delta I=I(t_{n}^{k}+{\bar{t}}_{n})-I(t_{n}^{k})\approx \delta \beta_{n}\frac{I(t_{n}^{k})[N_{n}(t_{n}^{k})-I(t_{n}^{k})]}{N_{n}(t_{n}^{k})}(t_{n}^{k}+{\bar{t}}_{n}-t_{n}^{k})$$ (16)

That is

$$Risk_{n}=\delta \beta_{n}\frac{I(t_{n}^{k})[N_{n}(t_{n}^{k})-I(t_{n}^{k})]}{N_{n}(t_{n}^{k})}{\bar{t}}_{n}$$ (17)

For $N_{n}(t_{n}^{k})$, $I(t_{n}^{k})$ in Eq (17) can be calculated as

$$N_{n}(t_{n}^{k})=\sum_{k\in K}\sum_{s\in S(n)}(1-\alpha_{s})q_{s}y_{ns}f_{n}^{k}$$ (18)

$$I_{n}(t_{n}^{k})=\sum_{k\in K}\sum_{s\in S(n)}(1-\alpha_{s}-{\hat{\alpha }}_{s})q_{s}y_{ns}f_{n}^{k}$$ (19)

where coefficients $\alpha_{s},\;{\hat{\alpha }}_{s}$ have been explained in Table 2.

In terms of Eqs (14), (17)–(19), we have the risk formula of each distribution center

$$Risk_{n}=\delta \beta_{n}\frac{\left[\sum_{k\in K}\sum_{s\in S(n)}(1-\alpha_{s}-{\hat{\alpha }}_{s})q_{s}y_{ns}f_{n}^{k}][\sum_{k\in K}\sum_{s\in S(n)}{\hat{\alpha }}_{s}q_{s}y_{ns}f_{n}^{k}\right]}{\sum_{k\in K}\sum_{s\in S(n)}(1-\alpha_{s})q_{s}y_{ns}f_{n}^{k}}\times \left[\sum_{i\in K}\sum_{s\in S}\frac{1}{v_{1}}(1-\alpha_{s})q_{s}y_{ns}f_{n}^{i}\right]$$ (20)

#### 3.3.3 Model design

According to the results in Section 3.3.1–3.3.2, the optimization model for the emergency routing problem considering the risk of virus infection is proposed as follows.


$$\begin{array}{l} \min TC=\sum_{k\in K}\sum_{m\in N}c_{k}^{V}x_{0m}^{k}+\sum_{w\in W}\sum_{s\in S}c_{w}^{U}p_{0s}^{w}+(c_{fuel}+c_{tax}\sigma_{V})\sum_{k\in K}\sum_{n\in N_{0}}\sum_{m\in N_{0}}x_{nm}^{k}\rho (Q_{n}^{k}-r_{n})d_{nm}\\ \;+(c_{fuel}+c_{tax}\sigma_{U})\sum_{w\in W}\sum_{i\in S_{0}}\sum_{j\in S_{0}}p_{ij}^{w}\mu (D_{i}^{w}-q_{i})d_{ij}+c_{r}\sum_{k\in K}\sum_{n\in N}t_{n}^{k}f_{n}^{k}\\ \;+\sum_{k\in K}\sum_{n\in N}\sum_{s\in S(n)}f_{n}^{k}\theta \max\{t_{n}^{k}+\frac{d_{sn}}{v_{walk}}+{\bar{t}}_{n}-LT_{s},0\} \end{array}$$ (21)

Subject to

$$Risk_{n}\leq R_{\max},\forall n\in N$$ (22)

$$y_{ns}=e_{s},\forall s\in S(n),n\in N$$ (23)

$$\sum_{s\in S(n)}q_{s}y_{ns}\leq E_{n},\forall n\in N$$ (24)

$$\sum_{s\in S(n)}q_{s}y_{ns}=r_{n},\forall n\in N$$ (25)

$$\sum_{k\in K}f_{n}^{k}=1,\forall n\in N$$ (26)

$$\sum_{\begin{array}{l} m\in N_{0}\\ m\neq n \end{array}}x_{mn}^{k}=f_{n}^{k},\forall n\in N,k\in K$$ (27)

$$\sum_{k\in K}\sum_{n\in N}x_{nm}^{k}=1,\forall m\in N_{0}$$ (28)

$$\sum_{n\in N_{0}}x_{nh}^{k}-\sum_{m\in N_{0}}x_{hm}^{k}=0,\forall h\in N,k\in K$$ (29)

$$Q_{0}^{k}=\sum_{n\in N}r_{n}f_{n}^{k}$$ (30)

$$Q_{m}^{k}=\sum_{n\in N_{0}}x_{nm}^{k}(Q_{n}^{k}-r_{n}),\forall m\in N$$ (31)

$$Q_{0}^{k}\leq \bar{Q^{k}},\forall k\in K$$ (32)

$$t_{n}^{k}+T_{n}+t_{nm}^{V}-t_{m}^{k}\leq (1-x_{nm}^{k})M$$ (33)

$$T_{n}=\max\{\sum_{i\in K}\sum_{s\in S(n)}\frac{(1-\alpha_{s})q_{s}}{v_{1}}y_{ns}f_{n}^{i},\sum_{i\in K}\sum_{s\in S(n)}\frac{2d_{ns}\alpha_{s}q_{s}}{\varphi_{2}v_{2}}y_{ns}f_{n}^{i}\}$$ (34)

$$\sum_{w\in W}h_{s}^{w}=1-e_{s},\;\forall s\in S$$ (35)

$$\sum_{s\in S_{0}}p_{sj}^{w}=h_{j}^{w},\forall j\in S,w\in W$$ (36)

$$1-e_{j}\leq \sum_{s\in S}p_{0s}^{w}\leq 1,\forall w\in W,j\in S$$ (37)

$$1-e_{j}\leq \sum_{s\in S}p_{s0}^{w}\leq 1,\forall w\in W,j\in S$$ (38)

$$\sum_{j\in S_{0}}p_{sj}^{w}-\sum_{j\in S_{0}}p_{js}^{w}=0,\forall s\in S,w\in W$$ (39)

$$\sum_{s\in S_{0}}\sum_{w\in W}p_{sj}^{w}=1-e_{j},\forall j\in S$$ (40)

$$D_{0}^{w}=\sum_{s\in S}q_{s}h_{s}^{w}$$ (41)

$$D_{s}^{w}=\sum_{j\in S_{0}}p_{js}^{w}(D_{j}^{w}-q_{s}),\forall s\in S$$ (42)

$$D_{0}^{w}\leq \bar{D},\forall w\in W$$ (43)

$$t_{s}^{w}+T_{s}+t_{sj}^{U}-t_{j}^{w}\leq (1-p_{sj}^{w})M$$ (44)

$$t_{sj}^{U}=\underset{w\in W}{\sum }\underset{s\in S}{\sum }\frac{q_{s}d_{sj}}{\varphi_{3}v_{3}}h_{sw}$$ (45)

In this model, the objective function (21) aims to minimize the sum of fixed cost, green cost, refrigeration cost and delay penalty cost, as shown in Eqs (1,11–13). Eq (22) represents a constraint that the risk of each distribution center, as shown in Eq (20), cannot exceed upper limit *R*_max_. Note that *R*_max_ is the acceptable upper limit for the number of new infections caused by residents gathering at a distribution center to pick up goods. Undoubtedly, there will be no new infection if all residents are serviced by large UAVs. However, the large UAV has a much higher operation cost than the vehicle and the quantity of large UAVs is usually very limited. Therefore, the logistics company has to maintain a balance between the use of vehicles and large UAVs. The cost is that, when trucks arrive at one distribution center and unload the goods, the residents have to gather at the center to pick up their goods, resulting in more infections due to direct close contact. Eq (23) means that distribution center *n* will service its own housing estate *s* (*y*_*ns*_ = 1) if *s* is not serviced by large UAVs (*e*_*s*_ = 1). Eq (24) implies that the goods shipping to a distribution center cannot exceed its capacity. Eq (25) the demand of every housing estate must be satisfied. Eq (26) sets that each distribution center can be serviced by only one vehicle. Eq (27) guarantees that a vehicle must travel to a center if the vehicle aims to service it. Eq (28) indicates that each vehicle can pass through a distribution center only once. Eq (29) means that any vehicle cannot stop at each distribution center finally. Eqs (30–31) are computational formulas of $Q_{0}^{k},Q_{m}^{k}$, the goods weight of a vehicle just arriving at a distribution center. Eq (32) represents that the goods weight of a vehicle starting from the logistics center cannot exceeds its capacity. Eqs (33, 44) are the constrains of subtour-elimination for vehicles and large UAVs, respectively. Eq (34) is the formula of the waiting time the vehicle needs at a center, where *v*_1_ is the average pick-up speed of residents, *v*_2_ is the flying speed of small UAVs and *φ*_2_ is the total capacity of all small UAVs for a vehicle. Eq (35) indicates that a large UAV team is assigned to service a housing estate only if the estate is not serviced by any vehicle. Eq (36) implies that a large UVA team can fly to a housing estate only if the team has been assigned to service the estate. Eq (39) sets that any large UAV team cannot stop at any housing estate finally. Eqs (40–41) are computational formulas of $D_{0}^{w},D_{s}^{w}$, the goods weight of a team of large UAVs just arriving at a housing estate. Eq (43) represents that the goods weight of a large UAV team starting from the logistics center cannot exceed its capacity. Eq (45) is the formula of the waiting time the team of large UAVs needs at a housing estate, where *v*_3_ is the flying speed and *φ*_3_ is the total capacity of a large UAV team.

Particularly, there is an obvious overlap of service object between the vehicle and the large UAV. For example, once one housing estate has been serviced by a team of large UAVs, the related distribution center nearby will not provide the goods to this housing estate. And the goods of distribution center are delivered by vehicles.

## 4. Solution algorithm

In this section, a modified ant colony algorithm (MACO) is used to solve the new model, which belongs to the NP-Hard problem. It is well known that NP-Hard problems are often solved by intelligence algorithms, such as genetic algorithm (GA) [15], ant colony algorithm (ACO) [42, 43] and simulated annealing algorithm (SA) [44]. Among these algorithms, ACO is well suited and effective. This is because the foraging routes of ants are highly similar to the transport routes of vehicles and drones in VPR/LPR, helping us flexibly improve ACO according to the characteristics of distribution routes of cars or drones.

However, many versions of ant colony algorithms (i.e. [15, 42, 43]) cannot be applied to our model directly because there is an obvious overlap of service object between the vehicle and the large UAV in our model. For the same reason, our model cannot be solved directly by some famous commercial solution software, such as CPLEX and GUROBI. To address this problem, we set two kinds of ants—one is the vehicle ant (VEA) and another one is the team of large UAV ant (UAA). The two kinds of ants look for food in rotation, every VEA only visits distribution centers directly and services related housing estates indirectly, and every UAA only visits housing estates and provides direct service.

Our modified ant colony algorithm (MACO) integrates the foraging routes of VEA and UAA, ensuring the flexible cooperation between the two types of ants. Then the overlapping issue of service objectives between cars and drones is solved. In the same iteration, specifically, for one VEA and one UAA, VEA first forages for food from the logistics center to some distribution center. For a candidate distribution center, there are several related housing estates. VEA must choose the housing estates which he should service if VEA decides to visit the distribution center. Meanwhile, VEA must avoid servicing any housing estate that has been serviced by some UAA. Once VEA has returned to the logistics center, UAA starts from the logistics center to some housing estate. Within the same iteration, UAA must avoid any housing estate which has ever been indirectly serviced by a vehicle. The above “VEA-UAA” cycle repeats until all housing estates have been serviced. After that, the VEA-pheromone and the UAA-pheromone are updated respectively, just like the classical ant colony algorithm [43]. Then this iteration ends and a new iteration starts.

According to the above discussion, we introduce the key details, subroutines and algorithm flow of MACO as follows [45].

### (1) Calculation of transition probability

In each iteration, the transition probability of an VEA $Ant_{k}^{VEA}$ visiting distribution center *j* from another distribution center *i* is

$$P_{ij}^{k}=\frac{\tau_{ij}^{\alpha }\eta_{ij}^{\beta }}{\sum_{l\in VN_{i}^{+}}\tau_{il}^{\alpha }\eta_{il}^{\beta }}$$ (46)

where $VN_{i}^{+}$ is the candidate set of the next feasible distribution center, *τ*_*ij*_ is the amount of pheromones, $\eta_{ij}={\left(Cost_{ij}^{V}\right)}^{-1}$ and the cost $Cost_{ij}^{V}$ of the VEA visiting from *i* to *j* is

$$Cost_{ij}^{V}=\left(c_{fuel}+c_{tax}\sigma_{V}\right)\rho \left(Q_{i}^{k}-r_{i}\right)d_{ij}+c_{r}t_{j}^{k}+\sum_{s\in S(j)}\theta \max\{t_{n}^{k}+\frac{d_{sj}}{v_{walk}}+{\bar{t}}_{j}-LT_{s},\;0\}$$ (47)

where the three terms on the right-hand side of Eq (47) represent the green cost, the refrigeration cost and the delay penalty cost, which are derived in Eqs (8), (12) and (13), respectively. Similarly, the transition probability of an UAA $Ant_{s}^{UAA}$ visiting housing estate *j* from another housing estate *i* is

$$\gamma_{ij}^{k}=\frac{\xi_{ij}^{\alpha }\rho_{ij}^{\beta }}{\sum_{l\in UN_{i}^{+}}\xi_{il}^{\alpha }\rho_{il}^{\beta }}$$ (48)

where $UN_{i}^{+}$ is the candidate set of the next feasible housing estate, *ξ*_*ij*_ is the amount of pheromones, $\rho_{ij}={\left(Cost_{ij}^{U}\right)}^{-1}$ and the cost $Cost_{ij}^{U}$ of the UAA visiting from *i* to *j* is

$$Cost_{ij}^{U}=(c_{fuel}+c_{tax}\sigma_{U})\mu (D_{i}^{w}-q_{i})d_{ij}$$ (49)

which is derived in Eq (9).

### (2) Updating strategy of pheromone

At first, the pheromone *τ*_*ij*_ of VEA $Ant_{s}^{UAA}$ in each iteration is updated by

$$\tau_{ij}^{new}=\rho \tau_{ij}^{old}+\Delta \tau_{ij}$$ (50)

$$\Delta \tau_{ij}=\sum_{k}\frac{Q}{TD_{k}^{VEA}}$$ (51)

where *ρ*∈(0,1), *Q*>0 and $TD_{k}^{VEA}$ is the length of the complete path (from the logistics center to the center itself) that the *k-th* VEA $Ant_{k}^{VEA}$ has built. Second, the pheromone *ξ*_*ij*_ of UAA $TD_{k}^{UAA}$ is updated by

$$\xi_{ij}^{new}=\rho \xi_{ij}^{old}+\Delta \xi_{ij}$$ (52)

$$\Delta \xi_{ij}=\sum_{k}\frac{Q}{TD_{k}^{UAA}}$$ (53)

where *ρ*∈(0,1), *Q*>0 and $TD_{k}^{UAA}$ is the length of the complete path (from the logistics center to the center itself) that the *k-th* UAA $Ant_{s}^{UAA}$ has built.

### (3) Choice of the feasible service object in the region of *j* for VEA

Suppose one VEA $Ant_{k}^{VEA}$ has reached distribution center *i* and starts to evaluate another distribution center *j*. Denote the set of housing estates belonging to the scope of service of distribution center *j* as *S*(*j*) = {*s*_1_,*s*_2_,⋯,*s*_*G*_}. $Ant_{k}^{VEA}$ selects the housing estates in the region of *j* to service according to the following procedure (Note that the service object for VEA is housing estates, not distribution centers).

*Step 1*: Set *k = 1* and the set of feasible service objects *F*(*j*) = {}.

*Step 2*: For *s*_*k*_∈*S*(*j*), if *S*_*k*_ has not been serviced by VEA or UAA in the same iteration, go to the next step; otherwise, go to *Step 5*.

*Step 3*: If $Q_{i}^{k}-r_{i}-q_{s_{k}}>0$, go to the next step; otherwise, go to *Step 5*.

*Step 4*: Suppose that $Ant_{k}^{VEA}$ visits from *i* to *j* and services the housing estates in *F*(*j*)∪{*s*_*k*_}. If *Risk*_*j*_≤*R*_*max*_ according to Eq (20), set *F*(*j*) = *F*(*j*)∪{*s*_*k*_} and $j\in VN_{i}^{+}$ ($VN_{i}^{+}$ is the set of the feasible visiting point for $Ant_{k}^{VEA}$).

*Step 5*: Set *k = k+1*. If *k*>*G*, stop and output *F*(*j*) (the set of feasible service objects for $Ant_{k}^{VEA}$); otherwise, go to *Step 2*.

In the above procedure, $Q_{i}^{k}-r_{i}-q_{s_{k}}>0$ in *Step 3* means that $Ant_{k}^{VEA}$ has enough goods to meet the demand of housing estate *s*_*k*_ after visiting distribution center *i*, *Risk*_*j*_≤*R*_max_ in *Step 4* implies that the risk can be controlled if *s*_*k*_ is added to the set of service object of $Ant_{k}^{VEA}$, i.e. *s*_*k*_ can be set as one feasible service object of $Ant_{k}^{VEA}$.

### (4) Choice of the feasible service object for UAA

Suppose that one UAA $Ant_{k}^{UAA}$ has reached housing estate *i*. Denote *H* = {*j*_1_,*j*_2_,⋯,*j*_*h*_} as the set of housing estates which have not been serviced by VEA or UAA in the same iteration. $Ant_{k}^{UAA}$ constructs the set of feasible service objects $UN_{i}^{+}$ based on the following procedure.

*Step 1*: Set *k = 1* and the set of feasible service objects $UN_{i}^{+}=\{\}$.

*Step 2*: For *j*_*k*_∈*H*, if $D_{i}^{w}-q_{i}-q_{j_{k}}>0$, set $UN_{i}^{+}=UN_{i}^{+}\cup \left\{j_{k}\right\}$.

*Step 3*: Set *k = k+1*. If *k*>*h*, stop and output $UN_{i}^{+}$; otherwise, go to *Step 2*.

In the above procedure, $D_{i}^{w}-q_{i}-q_{j_{k}}>0$ in *Step 2* means that $Ant_{k}^{UAA}$ has enough goods to meet the demand of housing estate *j*_*k*_ after visiting housing estate *i*, then housing estate *j*_*k*_ is included in $UN_{i}^{+}$.

### (5) Stopping criterion of MACO

In this paper, MACO will be terminated if one of the following criteria is satisfied:

1. Stop if no better solution can be generated in consecutive *L* iterations (*L* is a pre-defined counter value).
2. Stop if maximum iterations *MAXGEN* is reached.

### Algorithm flow of MACO

*Step 0*. Set *K* VEAs and *K* UAAs, the maximum number of iterations *MAXGEN*, the maximum number of consecutive iterations that can be tolerated if no better solution is produced *L*, iteration number *gen = 0*.

*Step 1*. If stopping criterion procedure (5) is satisfied, stop; otherwise, set *gen = gen+1*, go to the next step.

*Step 2*. For *k = 1*, *2*,…, *K*,

*Step 2*.*1*. The k-th VEA $Ant_{k}^{VEA}$ leaves the logistics center *0* and visits a series of distribution centers gradually and services a series of housing estates. The operations of collecting the feasible service objects and feasible visiting center for $Ant_{k}^{VEA}$ are conducted by procedure (3). The operations of determining the next visiting center and related service objects for $Ant_{k}^{VEA}$ are conducted by procedure (1). When $Ant_{k}^{VEA}$ returns to the logistics center *0* and builds a complete path $Path_{k}^{VEA}$, go to *Step 2*.*2*.

*Step 2*.*2*. The k-th UAA $Ant_{k}^{UAA}$ leaves the logistics center 0 and visits a series of housing estates gradually. The operations of collecting the feasible service objects, i.e. the feasible visiting center, are implemented by procedure (4). The operations of determining the next visiting housing estate are implemented by procedure (1). When $Ant_{k}^{UAA}$ returns to the logistics center *0* and builds a complete path $Path_{k}^{UAA}$, go to *Step 2*.*3*.

*Step 2*.*3*. For the “Vehicle-UAV” joint distribution scheme $Path_{k}^{VEA}+Path_{k}^{UAA}$, calculate the total cost *Total*_cos*t*_*k*_.

*Step 3*. Select the “Vehicle-UAV” distribution scheme with the minimum total cost from cost set {*Total*_cos*t*_*k*_; *k* = 1,2,⋯*K*}.

*Step 4*. Update the pheromone of VEA and UAA according to procedure (2). Set *gen = gen+1*, go to *Step 1*.

## 5. Numerical studies

In this section, we first test the effectiveness of the modified ant colony algorithm (MACO) proposed in Section 4. Then the distribution data of a cold chain Logistics Company is used to verify the model and obtain useful results.

### 5.1 Algorithm experiment

In this part, the genetic algorithm (GA), the simulated annealing algorithm (SA) and MACO are used to solve the model of Section 3.3.3.

The test data is called as *n-m-k*, where *n* represents the number of housing estates, *m* represents the number of distribution centers, *k* represents the location distribution rule of housing estates. The locations of housing estates are uniformly distributed if *k = 1*; randomly distributed if *k = 2*; half-and-half if *k = 3*. The unique logistics center is randomly distributed. Because each housing estate can be serviced by at most one distribution center, it is necessary to determine the locations of distribution centers. To do so, we use the square grid to divide the whole plane into several regions. Then the centre-of-gravity method is used to calculate the location of the unique distribution center corresponding to the distribution centers within each region. It is set that the capacity of each distribution center is 150 tons, the total demand of each housing estate is 1500kg, every family needs 5 kg goods and the ratio of infected residents who are sent home and put in quarantine is 0.05. In addition, maximum acceptable risk *R*_max_ = 30 and overall infection rate of each distribution center *δβ*_*n*_ = 0.1. The parameter settings of these algorithms do have obvious effects on the solution quality. Thus, we first refer to the relevant literature [15, 42–45] which focused on the intelligent optimization algorithms for solving LPR. According to the above literature, we set the maximum iterations is 300 for GA and MACO. The other parameters are set as: (1) For GA, population size *Np* = 100, crossover probability *p*_*c*_ = 0.8, and mutation probability *p*_*m*_ = 0.2; (2) For SA, initial temperature *T*_0_ = 100, temperature cooling coefficient α = 0.95, and termination temperature *T*_*f*_ = 0.05; (3) For MACO, number of ants *N* = 100, pheromone importance factor *α* = 3, heuristic importance factor *β* = 2, pheromone evaporation factor *ρ* = 0.2, and total pheromone deposit Q = 500. Based on the above settings, the solution quality can be guaranteed in the following experiments.

**Fig 3:**
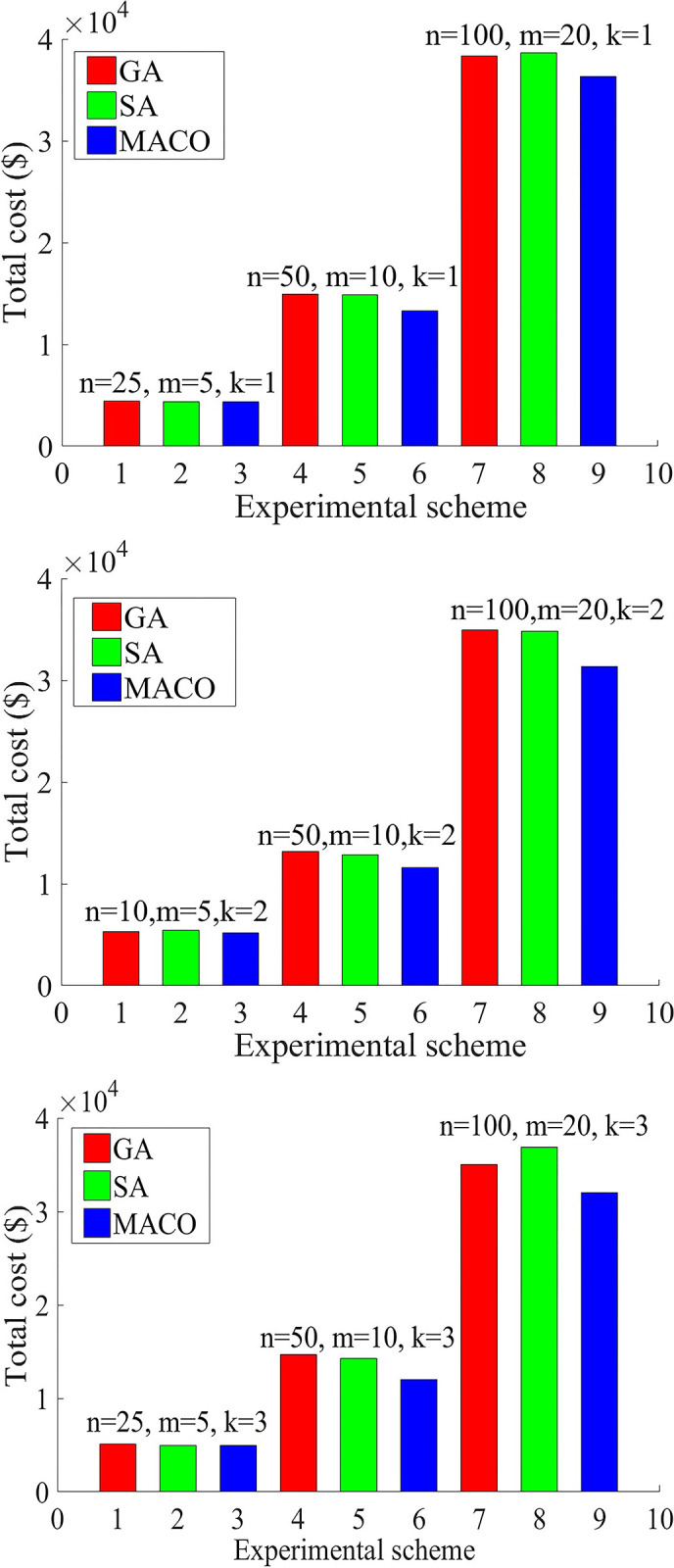
Comparison of GA, SA and MACO in reducing total cost under different experimental schemes.

The results are shown in Table 3 and Fig 3, where *t* is the time spent at the end of the algorithm.

**Table 3:** Experimental results of MACO and other algorithms.

Example			GA		SA		MACO	
*n*	*m*	*k*	Cost ($)	*t*	Cost ($)	*t*	Cost ($)	*t*
25	5	1	4440.50	5.20	4339.95	5.40	4350.47	5.50
25	5	2	5289.02	5.20	5435.66	5.40	5198.84	5.60
25	5	3	5093.72	5.50	4981.36	5.40	4940.29	5.50
50	10	1	14961.33	20.60	14856.10	20.50	13273.03	19.50
50	10	2	13205.32	21.30	12873.42	20.80	11586.01	19.50
50	10	3	14681.07	21.20	14305.04	20.70	12053.39	20.00
100	20	1	38309.84	108.70	38634.47	109.60	36313.81	99.40
100	20	2	34908.45	109.00	34791.33	109.50	31359.75	98.60
100	20	3	35025.83	109.30	36851.24	109.60	32004.62	98.70

As shown in Table 3 and Fig 3, for the solutions of the above 9 examples, MACO always has a certain advantage over GA and SA in terms of reducing total cost of the model. According to the computation time, MACO is superior to GA and SA in more than 77% of cases. Therefore, the MACO is competitive in solving our model in Section 3.3.3.

### 5.2 Model experiment

**Fig 4:**
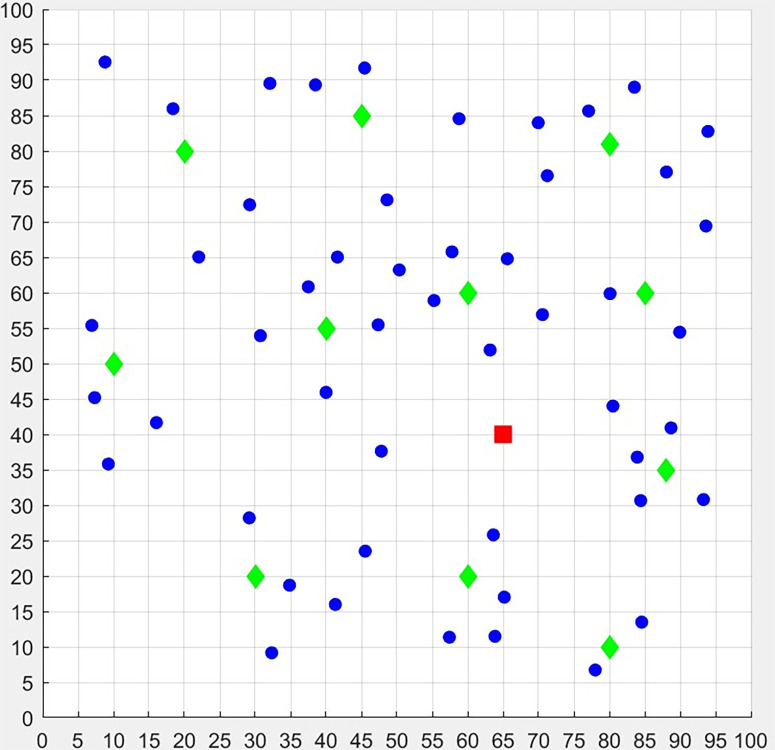
The locations of the unique logistics center (red), 11 distribution centers (green) and 50 housing estates (blue).

In this section, the distribution data of a cold chain Logistics Company is used to verify the model and obtain useful results. The company mainly provides warehousing and distribution services for refrigerated foods, such as dairy products, chilled meat, and so on. It has the only one logistics center and 11 candidate distribution centers in a certain area with a capacity of 150 tons (the locations are shown in Fig 4). Before arranging a delivery task, a total of 50 orders of demand points (housing estates) is received. The specific information such as the geographical location, latest service demand time, and demands are shown in S1 Appendix. Every family needs 5 kg goods and sends one person to take delivery of goods, if the family is serviced by a distribution center.

**Fig 5:**
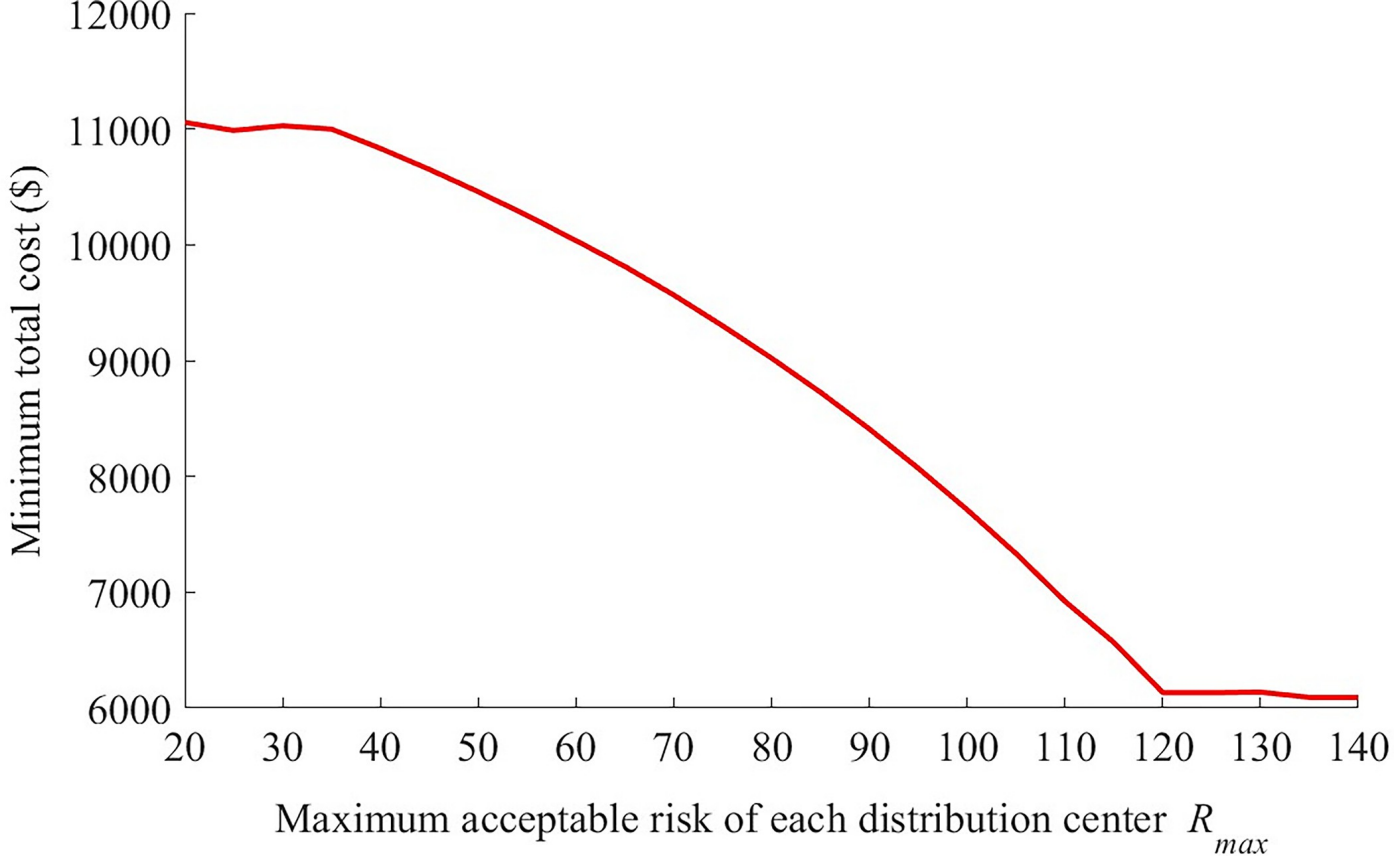
The change of minimum total cost with different values of *R*_max_ (*δβ*_*n*_ = 0.1).

**Fig 6:**
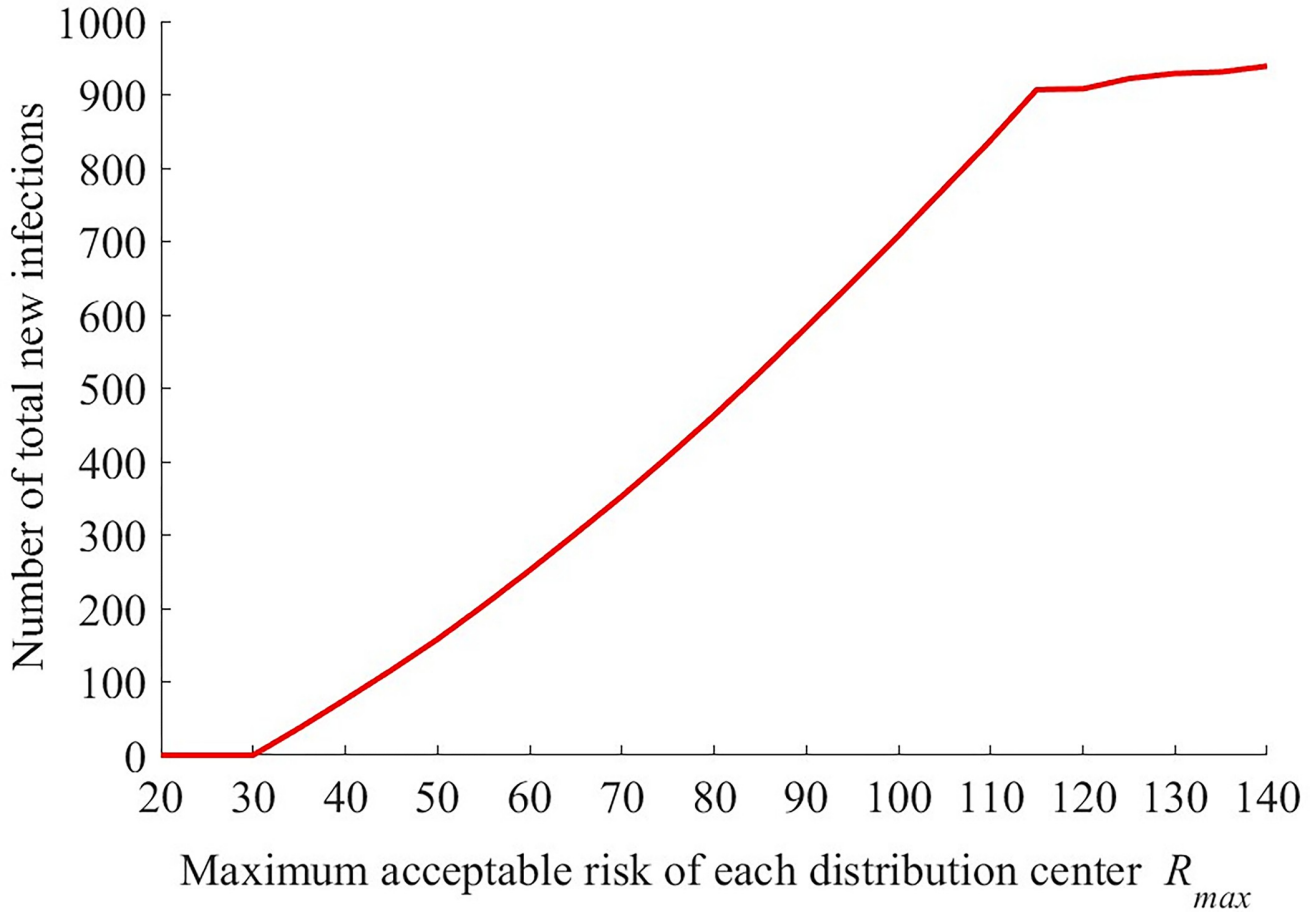
The change of number of new infections with different values of *R*_max_ (*δβ*_*n*_ = 0.1).

**Fig 7:**
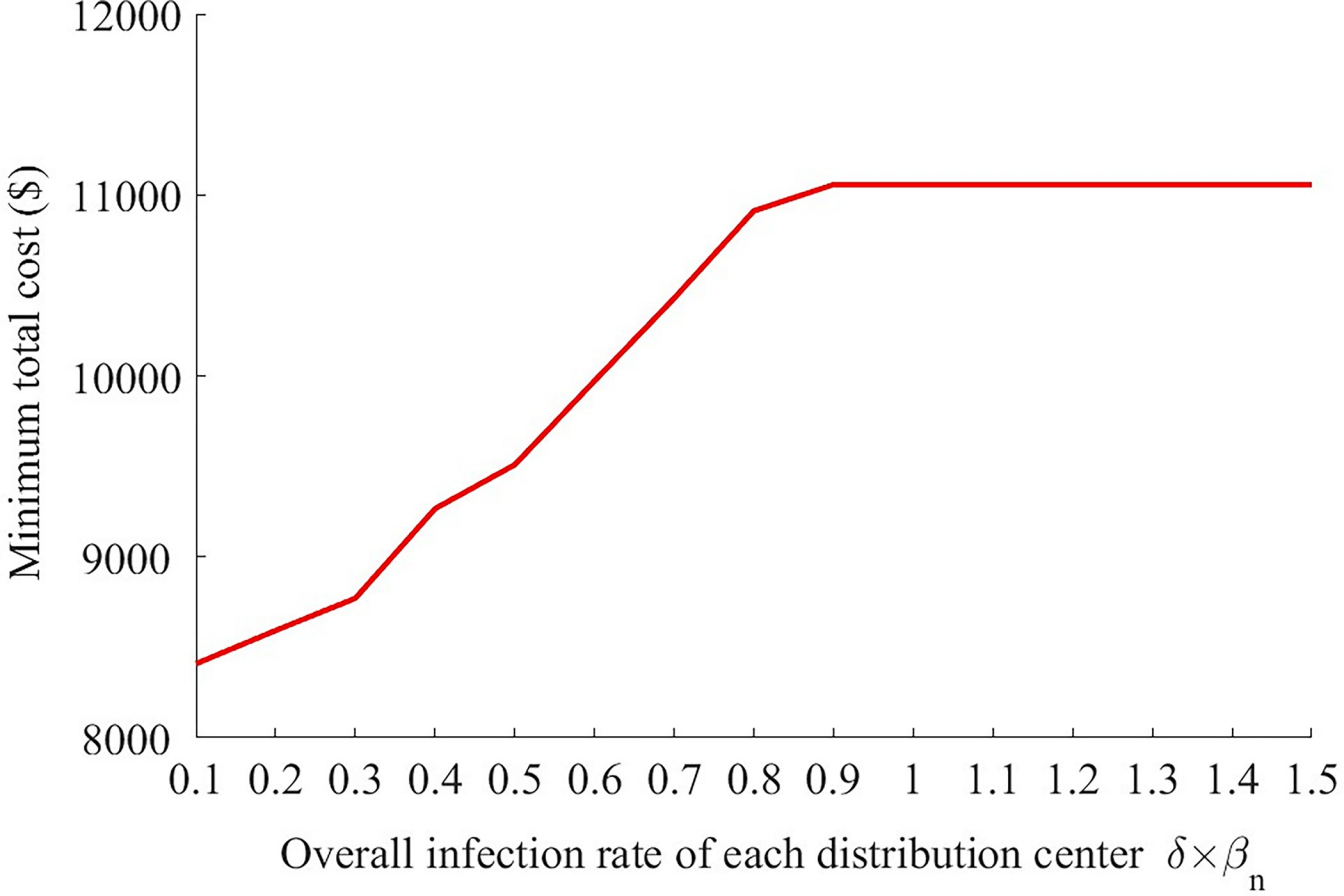
The change of minimum total cost with different overall infection rates *δβ*_*n*_ (*R*_max_ = 80).

**Fig 8:**
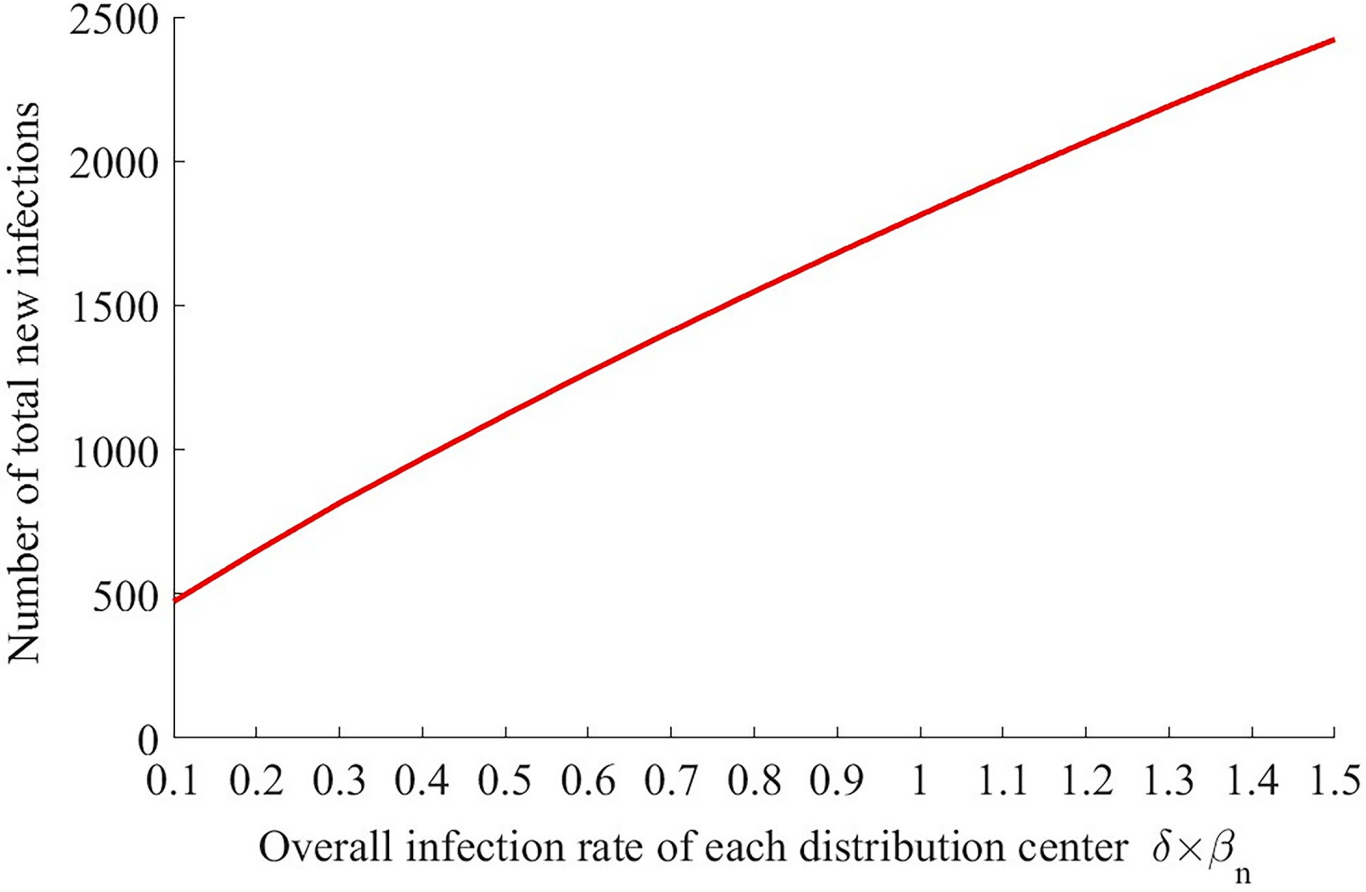
The change of number of new infections with different overall infection rates *δβ*_*n*_ (*R*_max_ = 80).

The effects of risk constraint *R*_max_ and overall infection rates *δβ*_*n*_ on the minimum total cost are presented in Figs 5–8.

The minimum total cost changes little when *R*_max_ increases from 20 to 30, decreases rapidly with *R*_max_ if *R*_max_ falls into interval *[40 120]*, then remains stable if *R*_max_ is larger than 120. In fact, a higher value of risk constraint means that more infections, as well as more residents getting together at the distribution center, are acceptable. And the large UAV has a higher unit cost than the vehicle. In order to reduce the total cost, as a result, the logistics company tends to replace the large UAVs with vehicles partly when *R*_max_ increases gradually. But this result is invalid if *R*_max_ is too small or large. Meanwhile, it can be seen from Fig 6 that, as *R*_max_ increases, the number of total new infections keep unchanged first, then increases quickly and increases slowly at last. Therefore, Figs 5 and 6 show that there is an obvious negative correlation between the minimum total cost of logistic company and the number of total new infections if *R*_max_ changes. That is to say, the decrease of *R*_max_ reduces the minimum total cost at the expense of the heath of residents.

As shown in Fig 7, the minimum total cost first increase quickly and then keeps unchanged as the overall infection rate of each distribution center *δβ*_*n*_ increases continuously. The cost reaches the highest level when *δβ*_*n*_ slightly increases to 0.9. Meanwhile, Fig 8 shows that the number of new infections also increases with *δβ*_*n*_ obviously. Note that a smaller value of *δβ*_*n*_ implies a higher management level. Thus, the improvement of management can reduce the total cost of logistic company and the number of new infections significantly at the same time. For the logistic company and the residents, the improvement of management of each distribution center is a win-win strategy, while another strategy—change of risk constraint presents a situation of “zero-sum game” (Fig 6). More specifically, *δβ*_*n*_ depends on *δ* (the infection rate when a healthy resident has close contact with one infector) and *β*_*n*_ (the average number of persons that a resident closely contacts when he is waiting to get the good). So, what the mangers of each distribution center should do is to persuade or even force every resident waiting at the center to wear masks (reduce *δ*) and keep distance with each other (reduce *β*_*n*_). For example, if everyone wears a mask, the infection rate when a healthy resident has close contact with one infector can reduce to 0.15%.

**Fig 9:**
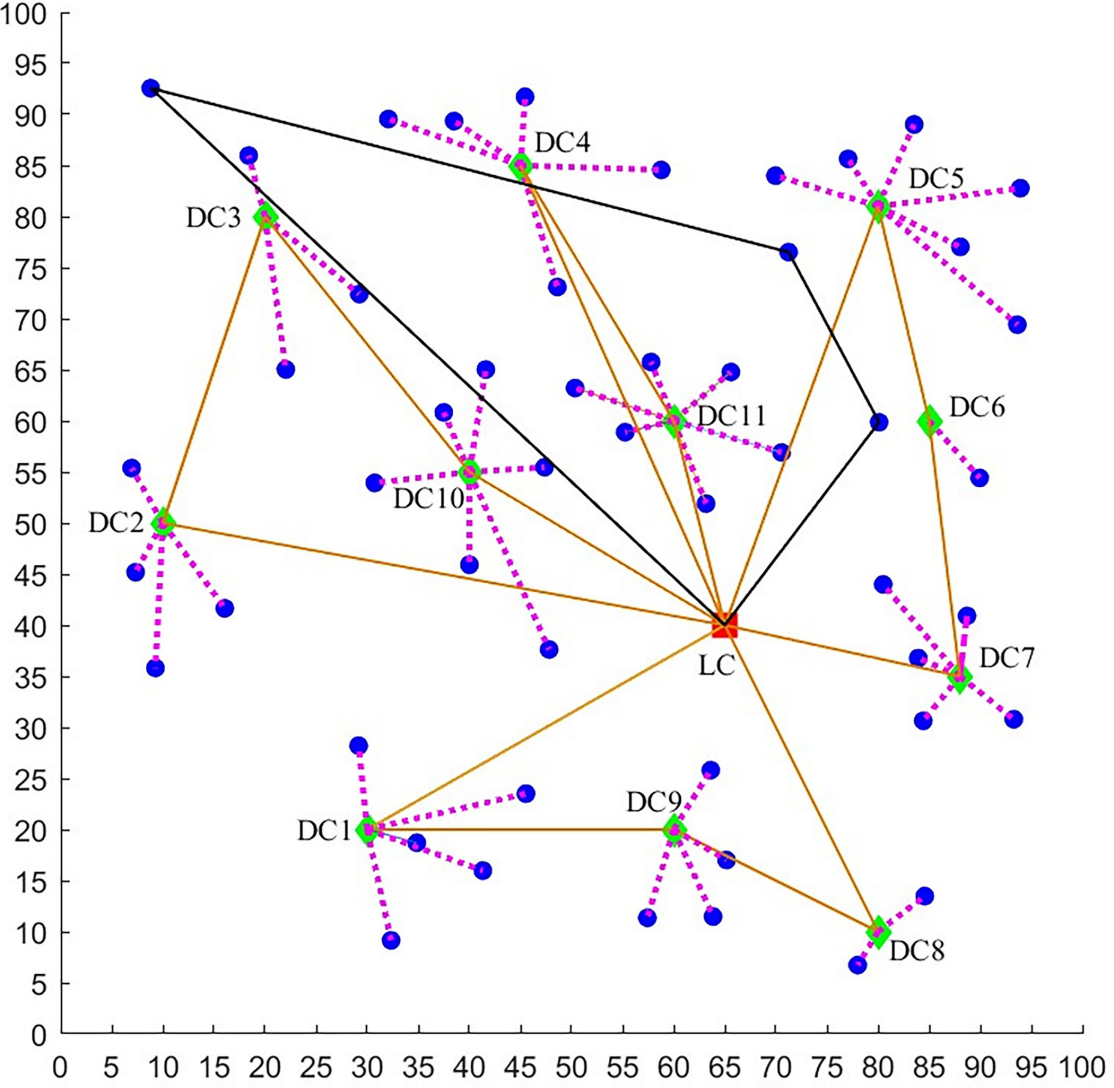
Distribution network with *R*_max_ = 140. (LC—logistics center, DC—distribution center, blue point—housing estate, rosy dashed line—the housing estate is serviced by a DC, golden solid line—vehicle path, **black solid line**—large UAV path).

**Fig 10:**
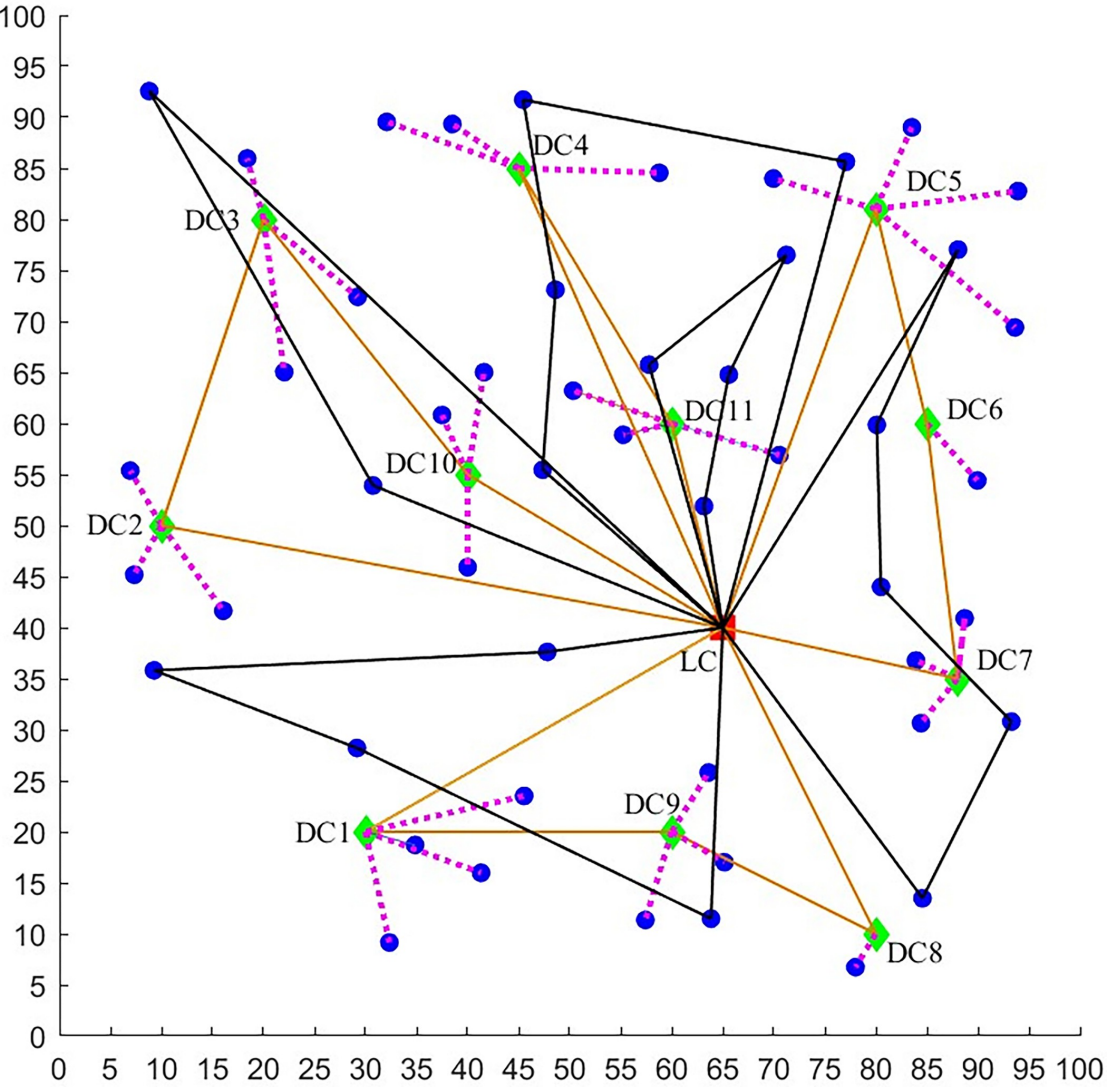
Distribution network with *R*_max_ = 70. (LC—logistics center, DC—distribution center, blue point—housing estate, rosy dashed line—the housing estate is serviced by a DC, golden solid line—vehicle path, **black solid line**—large UAV path).

In the following, we further explore the effect of risk constraint *R*_max_ on the distribution path scheme of vehicles and large UAVs. The distribution paths are compared between the high acceptable risk (*R*_max_ = 140) case and the moderate acceptable risk (*R*_max_ = 70), as shown by Figs 9 and 10 and Tables 4–6.

**Table 4:** The distribution routes with *R*_max_ = 140.

Route Number of vehicles	Distribution Service Order
1	LC-DC1-DC9-DC8-LC
2	LC-DC2-DC3-DC10-LC
3	LC-DC4-DC11-LC
4	LC-DC5-DC6-DC7-LC
Route Number of large UAV team	Distribution Service Order
1	LC-13-14-50-LC

**Table 5:** The distribution routes with *R*_max_ =70.

Route Number of vehicles	Distribution Service Order
1	LC-DC1-DC9-DC8-LC
2	LC-DC2-DC3-DC10-LC
3	LC-DC4-DC11-LC
4	LC-DC5-DC6-DC7-LC
Route Number of large UAV team	Distribution Service Order
1	LC-36-1-5-48-LC
2	LC-33-30-29-50-16-LC
3	LC-23-25-14-21-LC
4	LC-35-44-40-18-LC
5	LC-39-13-LC

**Table 6:** Comparison between *R*_max_ = 70 and *R*_max_ = 140.

*R*_max_	Number of Vehicles	Number of teams of large UAVs	Minimum total cost ($)
70	4	5	9097
140	4	1	6152

From the above results, we can see that *R*_max_ has a significant effect on the joint distribution scheme of large UAVs and vehicles. As *R*_max_ decreases from 140 to 70, the number of teams of large UAVs increases from 1 to 5 and the number of housing estates serviced by large UAVs increases from 3 to 19. Moreover, the vehicle distribution paths change obviously and the number of the housing estates serviced by vehicles decreases from 47 to 31. There is a positive correlation between the number of housing estates serviced by large UAVs and *R*_max_, and a negative correlation between the number of housing estates serviced by vehicles and *R*_max_. In addition, it is found that the number of vehicles and their distribution paths barely change as *R*_max_ increases, the only difference is the load of vehicles and the housing estates serviced.

### 5.3 Managerial implications

In summary, our model in this paper mainly aims to optimize the “Vehicle-UAV” joint distribution strategy of fresh goods during the epidemic, and the objective function is minimizing the total cost of a logistics enterprise based on considering the effect of home quarantine policy and controlling the risk of epidemic spreading. Thus, the sustainable development of society in the context of a major epidemic is truly realized. However, the total cost of considering the risk of epidemic spreading is obviously higher than the total cost when the risk is ignored for the design of distribution path optimization of cold chain logistics. This implies that it is necessary to pay a certain amount of economic costs to control the risk of epidemic spreading and realize the sustainable development of society towards today’s increasingly widespread threat of various infectious diseases.

From the governmental agencies’ point of view, first of all, what they should do is to pay attention to the risk issue of epidemic spreading during the logistics distribution process, strictly supervise the logistics enterprises and raise their awareness of this risk. Secondly, the local government must carefully assess the spatial distribution of the epidemic and available medical resources. On this basis, the government should set standards for epidemic prevention, i.e., the acceptable upper limit for the number of new infections per day, and negotiates with logistics companies on the proportion of drones and trucks used in the delivery process. Third, because the drone-delivery service does not result in more infections, the government can carry out subsidy policies and encourage enterprises to increase the proportion of drone deliveries to a certain extent.

From the cold chain logistics companies’ point of view, first of all, they should raise awareness of controlling the risk of epidemic spreading, strictly abide by the epidemic prevention policies implemented by the government and introduce the risk of cluster infection into the “Vehicle-UAV” joint distribution strategy optimization of cold chain logistics. As a result, the total cost and the number of new infections can be reduced simultaneously, and the demand of residents can also be satisfied timely. Second, the logistics enterprises must try hard to improve the management level of each distribution center because many residents gather there to get the goods. They need to effectively guide residents to wear masks and keep their distance to avoid close contact while waiting in line to pick up goods. Third, when epidemic breaks out, it may be not necessary for the logistics company to significantly change his daily vehicle distribution paths, he only needs to reduce the load of vehicles and sent more large UAVs to deliver the goods. Particularly, the paths of large UAVs must be updated.

When our model is applied in practice, there may be some operational, technical and regulatory challenges. For example, the cost of procurement, maintenance and use of drones, especially large drones, may be high; The joint delivery scheme requires the UAV to have accurate positioning function and real-time information sharing technology, and also requires the staff to be familiar with the standard operating technology of the UAV; In the process of joint distribution, the joint coordination and supervision of vehicles, small drones and large drones may have certain difficulties.

## 6. Conclusions and future research

This paper addresses the distribution routing problem for cold chain logistics during the pandemic, such as COVID-19 which spreads through the air. To control the risk of epidemic spreading, the home quarantine policy and an integrated “vehicle -UAV” transportation mode are introduced. In this case, the formula for the risk of epidemic spreading, which relates to the situation that residents get together at the distribution center to get the foods, is derived. Then the federated distribution network of vehicles and large UAVs is designed and a new logistics optimization model is developed.

Using a modified ant colony algorithm to solve the model, we study the effect of the maximum acceptable risk *R*_max_ and crowd management level of each distribution center in detail. The main conclusions are: (1) There is a negative correlation between the total cost of logistics company and the new infections, i.e., the decrease of *R*_max_ reduces the minimum total cost at the expense of the heath of residents. (2) the improvement of crowd management of distribution center reduces the total cost and the number of new infections significantly at the same time. (3) The maximum acceptable risk *R*_max_ has a significant effect on the distribution network and number of large UAVs. But this effect is not obvious for the paths of vehicles. This implies that the logistics company may not need to change his usual delivery paths of vehicles clearly when epidemic outbreaks. Instead, the company should reduce the load of vehicles, sent more large UAVs to deliver the goods and significantly updated the distribution network of large UAVs.

In the future, the development of artificial intelligence, Internet of Things, and 6G communication technology will further promote the effective operation of the "vehicle-drone" joint distribution model. For example, under the influence of the above technologies, multiple drones can realize real-time information sharing among each other, respond to the needs of residents in a more timely manner, and the drone group can automatically arrange distribution tasks and optimize the distribution network according to the needs of residents. As a result, distribution efficiency will be significantly improved.

Although our model and numerical results provide insights to the emergency logistics during the epidemic, there are still some limitation and problems which can be further considered. First, this paper only considers the example of a single logistics center, and the case of multiple logistics centers remains to be studied. Second, some uncertain events, such as the sudden intensification of the epidemic in a community, resulting in the failure of the previously specified distribution method to meet the demand, need to be further researched. A two-stage stochastic programming model with recourse should be developed. Finally, compared with the classical ant colony algorithms, our MACO algorithm has higher theoretical time/space complexity in one iteration because two kinds of foraging ants are set. In Section 4, we only use small and medium-sized numerical examples to test the effectiveness of MACO. Therefore, a numerical experiment based on real-world large-scale urban transportation networks is necessary for MACO in the future.

## Supporting information

S1 Appendix (DOCX)
