# Supplementary material for: Optimization of “vehicle-UAV” joint distribution routing for cold chain logistics considering risk of epidemic spreading and green cost
Source: PLoS One. 2024 Jun 26;19(6):e0306127. doi: 10.1371/journal.pone.0306127 (PMC11207173; doi:10.1371/journal.pone.0306127)
Supplement: S1 Appendix — (DOCX) [file pone.0306127.s001.docx]

**Appendix A.**

In this section, we provide the case study data that we have used to solve the presented model in Section 5.2. The numerical results are presented in Section 5.2.

**Table A1**

Demand information of each housing estate

| **Housing estate** | **X coordinate** | **Y coordinate** | **Total demand (kg)** | **Ratio of infected residents who are sent home and put in quarantine** $\alpha_{s}$ | **Percentage of infected residents who are asymptomatic and free to move** $\hat{\alpha}_{s}$ | **Latest service demand time** $LT_{s}$ |
| --- | --- | --- | --- | --- | --- | --- |
| 1 | 9.2661 | 35.8136 | 1800 | 0.05 | 0.01 | 2 |
| 2 | 6.9400 | 55.3857 | 1400 | 0.03 | 0.01 | 2.5 |
| 3 | 16.0533 | 41.6587 | 1900 | 0.05 | 0.01 | 2 |
| 4 | 7.3272 | 45.1878 | 2000 | 0.07 | 0.01 | 1.9 |
| 5 | 29.1595 | 28.2062 | 1900 | 0.03 | 0.01 | 2 |
| 6 | 34.8499 | 18.7011 | 1500 | 0.05 | 0.01 | 2.5 |
| 7 | 32.3295 | 9.1572 | 1300 | 0.05 | 0.01 | 1.8 |
| 8 | 45.5354 | 23.5105 | 1200 | 0.07 | 0.01 | 2 |
| 9 | 41.3142 | 15.9818 | 1300 | 0.05 | 0.01 | 2.5 |
| 10 | 22.0286 | 65.0408 | 2000 | 0.05 | 0.01 | 2.2 |
| 11 | 29.2182 | 72.4117 | 1300 | 0.03 | 0.01 | 2.3 |
| 12 | 18.3989 | 85.9742 | 1200 | 0.03 | 0.01 | 2.5 |
| 13 | 8.8068 | 92.5663 | 1600 | 0.07 | 0.01 | 3 |
| 14 | 71.2370 | 76.5214 | 2700 | 0.05 | 0.01 | 2.8 |
| 15 | 69.9578 | 84.0019 | 2200 | 0.05 | 0.01 | 2.1 |
| 16 | 88.0470 | 77.0335 | 2300 | 0.05 | 0.01 | 1.9 |
| 17 | 93.6299 | 69.4110 | 2100 | 0.07 | 0.01 | 2 |
| 18 | 77.0738 | 85.6500 | 1400 | 0.05 | 0.01 | 2.5 |
| 19 | 83.5297 | 89.0151 | 2000 | 0.03 | 0.01 | 2.7 |
| 20 | 93.9177 | 82.7733 | 1800 | 0.05 | 0.01 | 2.2 |
| 21 | 57.7796 | 65.7601 | 2500 | 0.05 | 0.01 | 2.5 |
| 22 | 50.3499 | 63.2129 | 2300 | 0.05 | 0.01 | 2 |
| 23 | 63.1672 | 51.9083 | 2200 | 0.03 | 0.01 | 1.9 |
| 24 | 55.2456 | 58.8981 | 2800 | 0.07 | 0.01 | 2.6 |
| 25 | 65.5903 | 64.7852 | 2900 | 0.05 | 0.01 | 3 |
| 26 | 70.5548 | 56.9082 | 2200 | 0.05 | 0.01 | 2.5 |
| 27 | 83.9344 | 36.7828 | 1900 | 0.03 | 0.01 | 2 |
| 28 | 88.7037 | 40.9118 | 1600 | 0.07 | 0.01 | 2 |
| 29 | 80.5073 | 43.9935 | 2500 | 0.05 | 0.01 | 2 |
| 30 | 93.2813 | 30.7958 | 2100 | 0.05 | 0.01 | 2.4 |
| 31 | 84.4238 | 30.6455 | 2000 | 0.04 | 0.01 | 2.5 |
| 32 | 78.0036 | 6.7332 | 2200 | 0.05 | 0.01 | 2.3 |
| 33 | 84.5738 | 13.4851 | 2500 | 0.05 | 0.01 | 2 |
| 34 | 37.4920 | 60.8251 | 2000 | 0.06 | 0.01 | 1.8 |
| 35 | 47.3562 | 55.4642 | 2300 | 0.03 | 0.01 | 1.8 |
| 36 | 47.8016 | 37.6213 | 1400 | 0.07 | 0.01 | 3.1 |
| 37 | 39.9995 | 45.9268 | 1900 | 0.05 | 0.01 | 2.6 |
| 38 | 41.6054 | 65.0238 | 1700 | 0.05 | 0.01 | 2.2 |
| 39 | 30.7352 | 53.9297 | 2800 | 0.05 | 0.01 | 2.2 |
| 40 | 45.4499 | 91.7177 | 2100 | 0.03 | 0.01 | 1.9 |
| 41 | 32.0737 | 89.5469 | 2000 | 0.08 | 0.01 | 2.7 |
| 42 | 58.7774 | 84.5615 | 1100 | 0.05 | 0.01 | 2.9 |
| 43 | 38.5081 | 89.3421 | 1400 | 0.05 | 0.01 | 2 |
| 44 | 48.6093 | 73.1074 | 2300 | 0.06 | 0.01 | 2.5 |
| 45 | 63.6215 | 25.8114 | 1200 | 0.05 | 0.01 | 2.3 |
| 46 | 57.4190 | 11.3616 | 1300 | 0.05 | 0.01 | 1.9 |
| 47 | 65.1661 | 17.0153 | 1100 | 0.07 | 0.01 | 3 |
| 48 | 63.8466 | 11.4846 | 2000 | 0.04 | 0.01 | 2.7 |
| 49 | 89.9358 | 54.4242 | 2000 | 0.05 | 0.01 | 2.5 |
| 50 | 80.0932 | 59.8667 | 1400 | 0.05 | 0.01 | 2.5 |

**Table A2**

Vehicle parameters

| Vehicle parameters | Parameter value |
| --- | --- |
| Capacity of vehicle $\overline{Q^{k}}$ | 30 ton |
| Vehicle speed $V_{e}$ | 60 km/h |
| Unit fixed cost of a vehicle  | 50 $ |
| Fuel price  | 1.1 $/kg |
| Carbon tax  | 1.0 $/kg |
| Fuel emission coefficient $\sigma_{i}$ | 2.63 kg/L |
| Unit refrigeration cost  | 1.5 $/h |
| Unit delay penalty cost  | 50 $/h |

**Table A3**

UAV parameters

| UAV parameters | Parameter value |
| --- | --- |
| Capacity of one team of large UAVs $\overline{D^{w}}$ | 10 ton |
| UAV speed $V_{UAV}$ | 150 km/h |
| Unit fixed cost of large UAV team  | 400 $ |
| Fuel price  | 1.1 $/kg |
| Carbon tax  | 1.0 $/kg |
| Fuel emission coefficient $\sigma_{i}$ | 2.63 kg/L |
